# Identification of key candidate genes and pathways in rheumatoid arthritis and osteoarthritis by integrated bioinformatical analysis

**DOI:** 10.3389/fgene.2023.1083615

**Published:** 2023-02-13

**Authors:** Huijing Huang, Xinyi Dong, Kaimin Mao, Wanwan Pan, Bin’en Nie, Lindi Jiang

**Affiliations:** ^1^ Department of Rheumatology, Zhongshan Hospital, Fudan University, Shanghai, China; ^2^ Shanghai Jiaotong University School of Medicine, Shanghai, China; ^3^ Department of Critical Care Medicine, Renji Hospital, School of Medicine, Shanghai Jiaotong University, Shanghai, China; ^4^ Yankuang New Journey General Hospital, Jingning, Shandong, China; ^5^ Department of Bone and Joint Surgery, Renji Hospital, School of Medicine, Shanghai Jiaotong University, Shanghai, China

**Keywords:** rheumatoid arthritis, osteoarthritis, microarray expression profiling dataset, differentially expressed genes, protein-protein interaction network

## Abstract

Rheumatoid arthritis (RA) and osteoarthritis (OA) are the most common joint disorders. Although they have shown analogous clinical manifestations, the pathogenesis of RA and OA are different. In this study, we used the online Gene Expression Omnibus (GEO) microarray expression profiling dataset GSE153015 to identify gene signatures between RA and OA joints. The relevant data on 8 subjects obtained from large joints of RA patients (RA-LJ), 8 subjects obtained from small joints of RA patients (RA-SJ), and 4 subjects with OA were investigated. Differentially expressed genes (DEGs) were screened. Functional enrichment analysis of DEGs including the Gene Ontology terms and Kyoto Encyclopedia of Genes and Genomes (KEGG) pathways were identified, which were mainly associated with T cell activation or chemokine activity. Besides, protein-protein interaction (PPI) network analysis was performed, and key modules were identified. Hub genes of RA-LJ and OA groups were screened, they were *CD8A*, *GZMB*, *CCL5*, *CD2,* and *CXCL9*, whereas *CD8A*, *CD2*, *IL7R*, *CD27,* and *GZMB* were hub genes of RA-SJ and OA group. The novel DEGs and functional pathways between RA and OA identified in this study may provide new insight into the underlying molecular mechanisms and therapeutic strategies of RA and OA.

## Introduction

According to recent studies, rheumatoid arthritis (RA) and osteoarthritis (OA) are considered to be the most prevalent rheumatic diseases, affecting 1% and 10% world’s population, respectively (van der Woude and van der Helm-van Mil, 2018). RA is an autoimmune disorder presented with chronic aggressive multiple arthritis and systemic manifestation. Metacarpophalangeal joints and wrists are the most commonly involved joints, followed by large joints like the knee (Smolen et al., 2016). The pathogenesis of RA has not yet been completely understood. However, it is generally acknowledged that genetic and environmental factors play important roles in this disease (McInnes and Schett, 2011). The majority of patients with RA suffer damage to their small joints in the inchoate stage (Nakajima et al., 2016). Recent treatment-to-target strategies using classical or biological disease-modifying anti-rheumatic drugs (bDMARDs) have allowed patients to achieve remission and have delayed progressive damage to their small joints (Smolen and Aletaha, 2015). A large joint (shoulder, elbow, hip, and knee) is usually destroyed during an advanced stage of RA. A patient with RA is not routinely monitored for progressive damage to large joints, even though damage to large joints has a considerably greater impact on functional ability than damage to smaller joints (Nakajima et al., 2016).

As the most common arthritis throughout the world, osteoarthritis is the leading cause of disability in the elderly (Abramoff and Caldera, 2020). OA destroys articular cartilage and stimulates the hyperplasia of the margin of bones as well as lesions in synovium and tissue of joints, which can lead to a series of biochemical and morphological changes in overused or weight-bearing joints unilaterally or bilaterally (Xia et al., 2014; Abramoff and Caldera, 2020). The pathogenesis of OA remains unclear. It is known that factors promoting inflammation, especially IL-1β and TNF-α play key roles in the development of OA. Several factors such as genetic predisposition, ageing, obesity, and joint misalignment have been implicated as contributing to the development of OA. A recent study by Butterfield et al. identified 14 genes involved in osteoarthritis pathogenesis, including *Pitx1*, and functionally characterize 6 candidate human osteoarthritis genes (*Unk, Josd1, Gsdme, Arhgap30, Ccdc6,* and *Col4a2*) in mouse models (Butterfield et al., 2021), revealing the genetic basis for osteoarthritis.

Although RA and OA share some clinical manifestations, they are two distinct arthritic disorders with RA being an autoimmune disease and OA being a degenerative disease (Vina and Kwoh, 2018). For identifying biomarkers for the diagnosis and prognosis of diseases, microarrays have become a promising and efficient tool for exploring significant genetic or epigenetic changes in disease (Cao et al., 2021b). RA patients and normal individuals, OA patients, and normal individuals as well as RA patients and OA patients, were found to have differentially expressed genes (DEGs) utilizing bioinformatic analysis (Lin et al., 2020; Triaille et al., 2020; Liu and Chen, 2021). Therefore, in this study, we compared gene expression profiles in synovial tissue between large and small joints of RA and joints of OA *via* bioinformatic analysis of an online dataset, seeking to identify possible key genes that involve the pathogenesis of joints of RA from joints of OA.

## Materials and methods

### Microarray data

The gene expression profile dataset GSE153015, deposited by Triaille C et al., was obtained from the Gene Expression Omnibus (GEO, https://www.ncbi.nlm.nih.gov/geo/) and was based on the platform of GPL570 Affymetrix Human Genome U133 Plus 2.0 Array [HGU133_Plus_2]. We collected samples from 24 subjects, including 10 small joints (metacarpophalangeal joints or wrists) from 10 RA patients, 10 large joints (knees) from the same 10 RA patients, and 4 OA subjects. The type of biological material used for gene expression quantification can be found in the study of Triaille C et al. (Triaille et al., 2020) Before the analysis, we did a principal component analysis (PCA) and found that two samples of large joints (LJ) of RA (GSM4633131, GSM4633133) and two samples of small joints (SJ) of RA group (GSM4633130, GSM4633135) were mixed with OA samples, respectively. As a result, we excluded the 4 samples to improve the quality of the samples. We obtained from the GEO dataset the age, gender, ACPA/RF status, DAS 28-CRP scores, and C reaction protein (CRP) levels of the individuals, as well as the annotation file for GPL570 and displayed in Table 1.

**TABLE 1:** Characteristics of the samples in this study.

Sample id	Diagnosis	Joint size	Age (year)	Gender	ACPA/RF positive	DAS28-CRP	CRP (mg/L)
GSM4633117	RA	LJ	36	F	Y	5.99	77
GSM4633119	RA	LJ	40	F	Y	6.22	44
GSM4633121	RA	LJ	60	F	Y	3.05	5
GSM4633123	RA	LJ	62	F	Y	4.54	1
GSM4633125	RA	LJ	78	F	Y	4.49	4
GSM4633127	RA	LJ	38	F	N	5.69	17
GSM4633129	RA	LJ	66	F	Y	4.27	14
GSM4633134	RA	LJ	30	F	Y	5.57	10
GSM4633116	RA	SJ	36	F	Y	5.99	77
GSM4633118	RA	SJ	40	F	Y	6.22	44
GSM4633120	RA	SJ	60	F	Y	3.05	5
GSM4633122	RA	SJ	62	F	Y	4.54	1
GSM4633124	RA	SJ	78	F	Y	4.49	4
GSM4633126	RA	SJ	38	F	N	5.69	17
GSM4633128	RA	SJ	66	F	Y	4.27	14
GSM4633132	RA	SJ	55	F	N	5.55	38
GSM4633136	OA	LJ	71	F	NA	NA	NA
GSM4633137	OA	LJ	77	F	NA	NA	NA
GSM4633138	OA	LJ	69	M	NA	NA	NA
GSM4633139	OA	LJ	66	F	NA	NA	NA

### Differential expression analysis

To screen for DEGs, we compared expression profiles of large and small joints of RA patients and OA patients using the online analysis tool GEO2R. *p*-values and adjusted *p*-values were calculated *via* t-tests and statistically significant DEGs were defined with the criteria of 1) a |log2 (fold-change)| >1 and 2) an adjusted *p* < 0.05. Patients were divided according to their joint size. RA patients with large joints were compared with RA patients with small joints. R software was used to draw the volcano plot and PCA, HTML software was used to create the heatmap for the DEGs, and Venn diagrams of DEGs were drawn using Venny 2.1 (https://bioinfogp.cnb.csic.es/tools/venny/index.html), and uniform manifold approximation and projection (UMAP) were performed *via* R’s “umap” package (Mao et al., 2020).

### Functional enrichment analysis of DEGs

Using Enrichr (https://amp.pharm.mssm.edu/Enrichr/), functional enrichment analyses of DEGs were conducted, including Gene Ontology (GO) terms and Kyoto Encyclopedia of Genes and Genomes (KEGG) pathways. A GO analysis consists of three biological processes (BP), a cellular component (CC), and a molecular function (MF), which provide a framework for describing the functions of gene products in all organisms. We assigned DEGs to specific pathways using KEGG pathway analysis (15). The threshold for significance was set at Benjamini-adjusted *p* < 0.05 and an enriched gene count of at least five genes enriched in the pathway.

### Protein-protein interaction (PPI) network construction

The PPI network analysis was carried out using Search Tool for the Retrieval of Interacting Genes (STRING, https://string-db.org/), a web-based database dedicated to predicting protein-protein interactions consisting of both physical and functional relations. We used Cytoscape v3.8.2 software to visualize and build the PPI network simultaneously while mapping the DEGs onto the PPI network with a medium confidence score of 0.4. Using ClusterOne from Cytoscape’s software suite, the gene network clustering analysis was conducted to refine the key PPI network modules. An R software package with the “ggplot2” package was used to analyze the expression levels of key PPI network modules (Cao et al., 2021a). The degree topological algorithm of Cytohubba was used to determine which nodes had the greatest number of interactions with neighboring nodes as hub genes.

### Immunohistochemistry

Paraffin joint tissue sections were cut into five mm-thick sections in the department of bone and joint surgery, at Renji hospital. Following deparaffinization and hydration, sections were treated with primary anti-CD8A antibodies (PA5-114369, Invitrogen), anti-CD2 antibodies (PA5-32312, Invitrogen), anti-GZMB antibodies (ab255598, Abcam), anti-CCL5 antibodies (ab52562, Abcam), anti-CXCL9 antibodies (ab290643, Abcam), anti-IL7R antibodies (ab259806, Abcam), and anti-CD27 antibodies (ab131254, Abcam). Incubation at room temperature for 30 min was followed by diluted biotinylated secondary antibodies. After incubation in Street Avidin-Biotin Complex for 20 min, sections were treated with diaminobenzidine (DAB) substrate solution until the desired color intensity was achieved (He et al., 2021; Mao et al., 2021). Images were taken under a light microscope (Nikon).

### Statistical analysis

To correct the *p*-value, the Benjamini–Hochberg FDR (false discovery rate) was used. Hypergeometric tests were used to distinguish significantly enriched GO terms and KEGG pathways. Statistical analysis of significant differences was conducted using one-way ANOVA by Prism 7 software. A *p*-value less than 0.05 was considered statistically significant.

## Results

### DEGs between RA and OA joints

**FIGURE 1:**
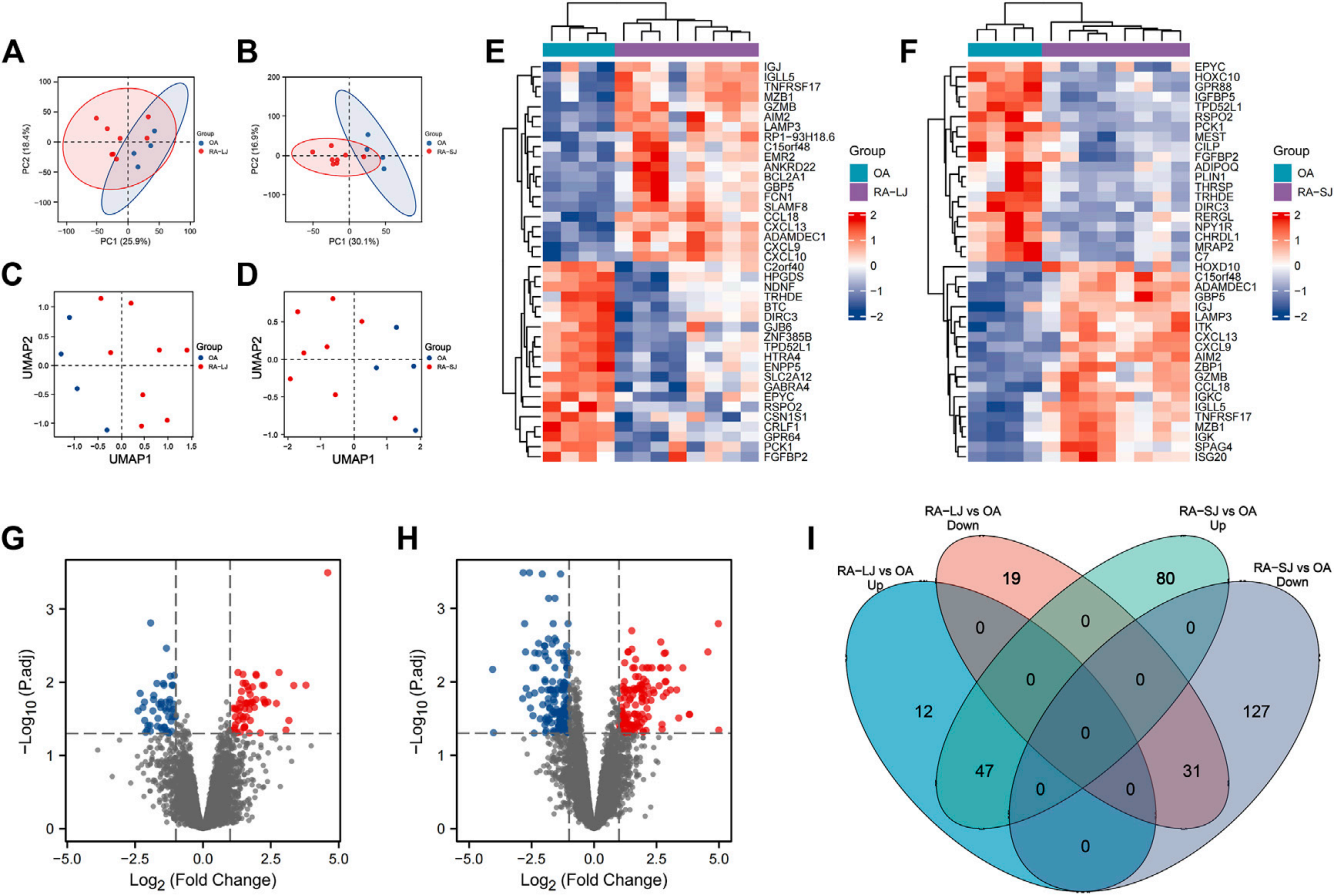
DEGs were identified from the online GEO dataset. **(A)**–**(D)** RA-LJ and RA-SJ samples are separated from OA samples in PCA and UMAP analyses. **(E) (F)** Heatmaps showed the top 20 upregulated and downregulated genes between RA and OA. **(G) (H)** Genes in RA samples and OA samples were significantly separated in the volcano plot. Red represents upregulated genes, and blue represents downregulated genes **(I)** Venn diagram of differentially expressed genes between RA-LJ and RA-SJ samples compared with OA samples.

The characteristics of the samples were listed in Table 1. The microarray expression dataset GSE153015 was downloaded from the GEO database, and DEGs were obtained between RA-LJ and OA (Supplementary Tables 1, 2). Based on the established criteria, we found that 59 genes were upregulated and 50 genes were downregulated between RA-LJ and OA, and 127 genes were upregulated and 158 genes were downregulated between RA-SJ and OA. According to the PCA and UMAP analyses in Figures 1A–D, the clusters of the two comparison groups were found in relatively independent quadrants, indicating that there was a significant difference in the RA-LJ and OA samples, as well as the RA-SJ and OA samples; Figures 1E, F show heatmaps for DEGs that show the top 20 upregulated genes in RA-LJ *versus* OA and RA-SJ *versus* OA, respectively; Figures 1G, H show volcano plots of compared groups. Our Venn diagram of the DEGs (Figure 1I) made it easier for the audience to understand the intersection and independence of the two DEG groups. According to the Venn diagram, 47 upregulated genes and 31 downregulated genes are shared by RA’s large and small joints. The overlapped genes made up 79.67% of upregulated genes in the RA-LJ group *versus* the OA group and 62% of downregulated genes in the RA-LJ group *versus* the OA group.

### Functional and pathway enrichment of DEGs

**FIGURE 2:**
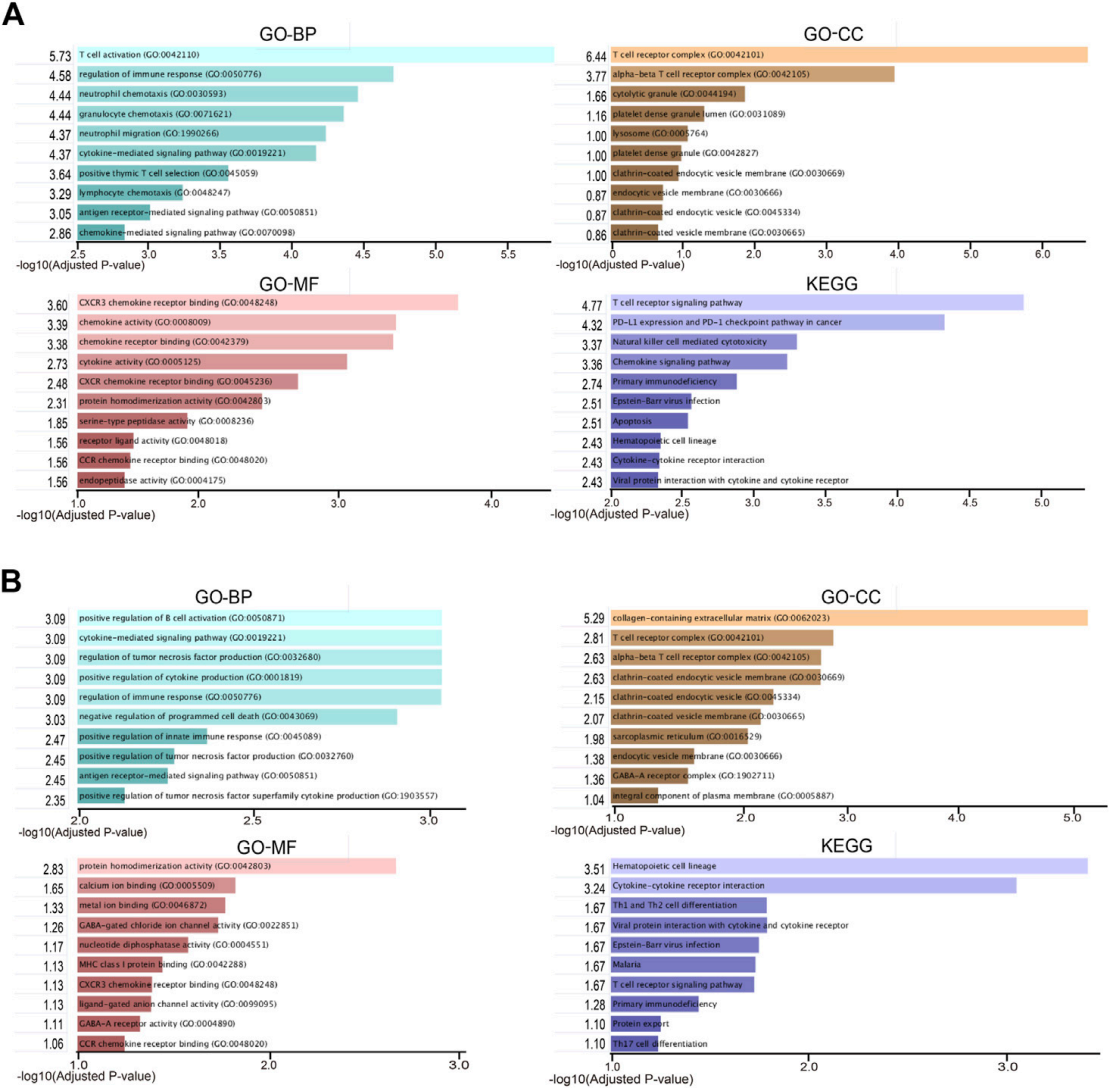
Representative enriched functional terms of RA samples compared with OA samples. **(A)** Most significantly enriched terms of GO-BP, MF, CC categories, and KEGG pathways in RA-LJ samples *versus* OA samples **(B)** Significantly enriched terms of GO-BP, MF, CC categories, and KEGG pathways in RA-SJ samples compared with OA samples. The *x*-axis represents the value of [-log (Adjusted *p*-value)].

To take a further step in the investigation of the biological functions of DEGs, a functional enrichment analysis was conducted and the results are presented in Figure 2. As a result, the DEGs in RA-LJ *versus* OA groups are mainly T cell receptor complexes in the CC category, chemokine receptor binding in the MF category, and T cell activation in the BP category. The T cell receptor signaling pathway is the most enriched KEGG pathway. The DEGs in RA-SJ compared to OA groups include collagen-containing extracellular matrix components, protein homodimerization activity in MF, and positive regulation of B cell activation and cytokine-mediated signaling pathways in BP. Pathways associated with hematopoietic cells were identified as the most significantly altered pathways in RA-SJ compared to OA.

### PPI network analysis of DEGs

**FIGURE 3:**
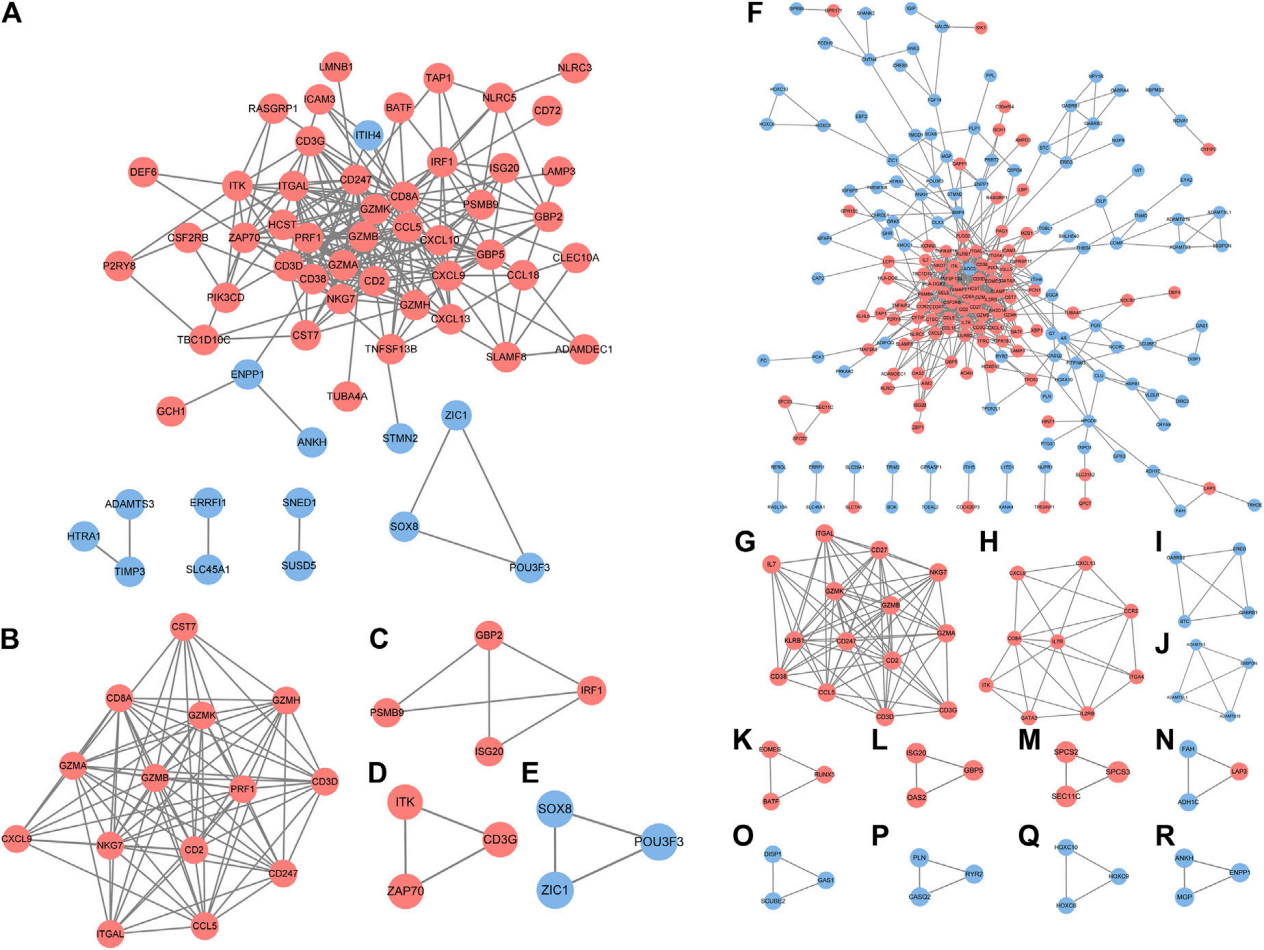
PPI network analysis of DEGs between RA-LJ/RA-SJ samples and OA samples. **(A)** Cytoscape network visualization of RA-LJ and OA samples that were obtained with interaction scores >0.4 according to the STRING online database **(B–E)** Four key modules were identified by ClusterOne. **(F)** Cytoscape network visualization of RA-SJ and OA samples **(G)**–**(R)** 12 key modules were identified by ClusterOne. The nodes represent genes, and the edges represent links between genes. Red represents upregulated genes, and blue represents downregulated genes.

In accordance with the STRING web-based database, two PPI networks were built with an interaction score >0.4 (Figure 3A). In order to identify the key PPI network modules, we performed network gene clustering in Cytoscape using ClusterOne, which corresponds to gene nodes and edges. In Figures 3B–E, a comparison between RA-LJ and OA reveals two key modules with 14 upregulated genes (*CST7, CD8A, GEMK, GZMH, GZMA, GZMB, PRF1, CD3D, CXCL9, NKG7, CD2, CD247, ITGAL, CCL5*) and three downregulated genes (*SOX8, ZIC1, and POU3F3*). While in Figures 3F–R, we identified 13 key modules with 33 upregulated genes (ITGAL, IL-7, CD27, CD38, GZMK, GZMA, GZMB, NKG7, KLRB1, CD247, CD2, CCL5, CD3D, CD3G, CXCL9, CXCL13, CD8A, IL7R, CCR2, ITK, GATA3, IL2RB, ITGA4, EOMES, BATF, RUNX3, ISG20, OAS2, GBP5, LAP3, SPCS2, SCPS3, SEC11C) and 22 downregulated genes (ADAMTSL1, ADAMTS3, ADAMTS16, SBSPON, GABRB1, GABRB2, BTC, EREG, HOXC6, HOXC9, HOXC10, DISP1, GAS1, SCUBE2, FAH, ADH1C, PLN, CASQ2, RYR2, ANKH, MGP, ENPP1) in RA-SJ *versus* OA.

**FIGURE 4:**
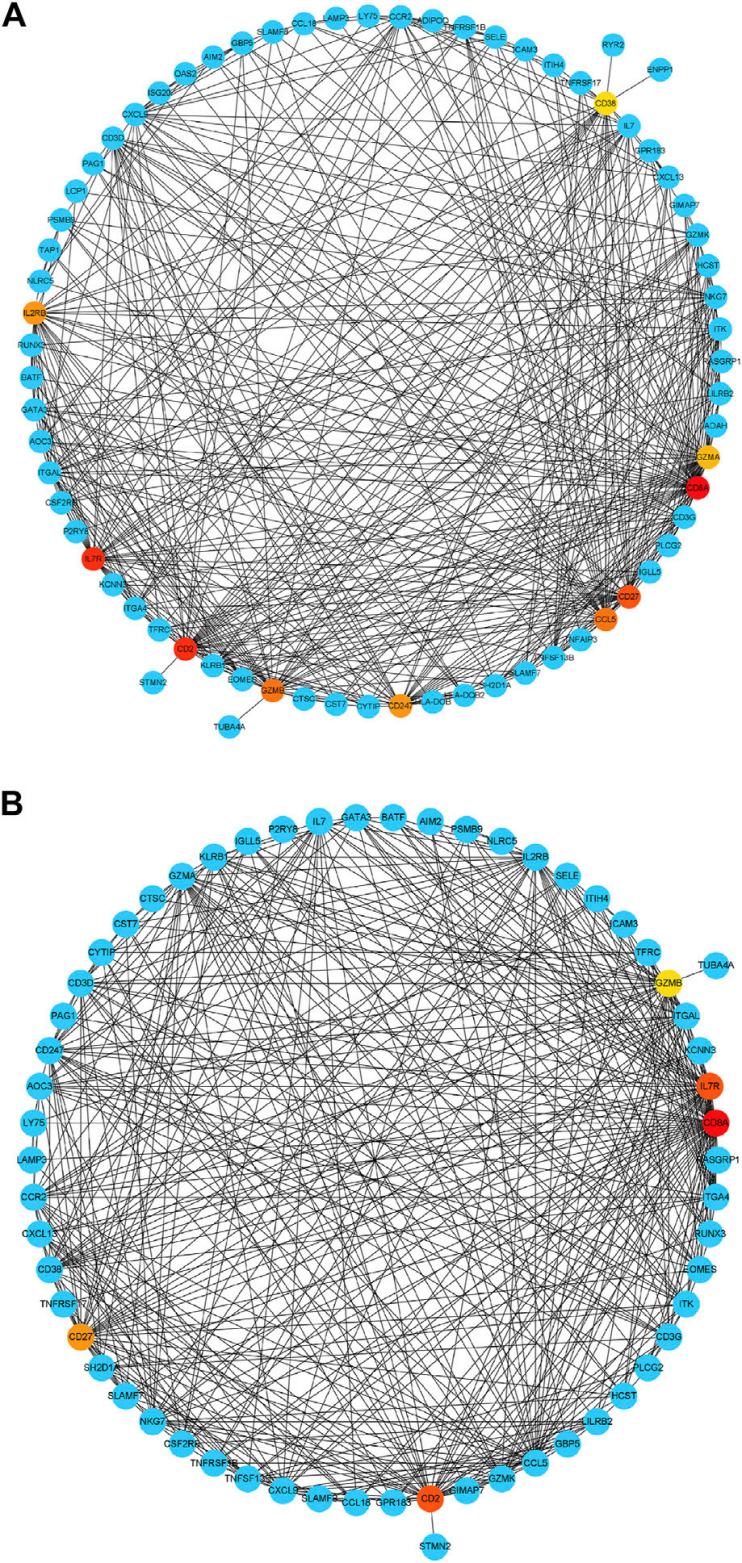
Detection of hub genes from the PPIs network of DEGs between RA and OA according to their degree value. **(A)** The highlighted five genes are *CD8A*, *GZMB*, *CCL5*, *CD2,* and *CXCL9* in RA-LJ samples compared with OA samples **(B)** The highlighted five genes are *CD8A*, *CD2*, *IL7R*, *CD27*, and *GZMB* in RA-SJ samples compared with OA samples. Red, orange and yellow modules represent hub genes, and the degree values of hub genes decrease from red to yellow modules. Blue modules refer to the genes which can interact with hub genes.

**FIGURE 5:**
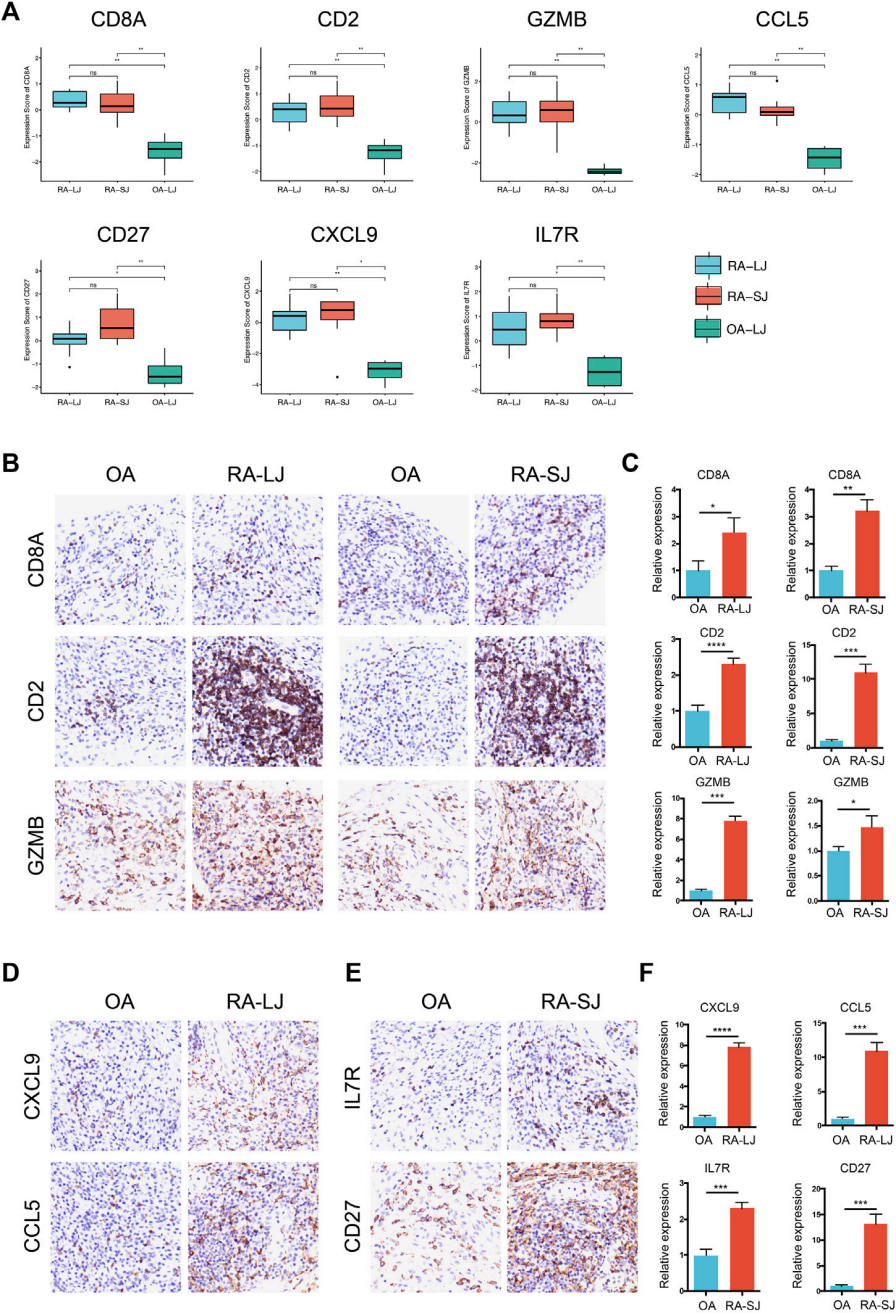
Expressions of hub genes in different groups in the microarray datasets and validations by immunohistochemical staining using joint biopsy sections. **(A)** Expressions of hub gene *CD8A*, *CD2*, *GZMB*, *CCL5*, *CXCL9*, *IL7R,* and *CD27* in RA-LJ, RA-SJ, and OA samples **(B)**–**(F)** The protein expressions of the 7 hub genes were determined by immunohistochemical staining using joint samples from patients. *p-value <0.05.**p-value <0.01. ***p-value <0.001. ****p-value <0.0001. Ns, no significance.

Based on their degree value, which represents the number of interactions among the genes, the hub genes were sorted. Figure 4A shows that the hub genes of the RA-LJ and OA groups are *CD8A, GZMB, CCL5, CD2,* and *CXCL9*, whereas Figure 4B shows the hub genes of RA-SJ and OA groups as *CD8A, CD2, IL7R, CD27,* and GZMB. Among them, *CD8A, GZMB,* and *CD2* are hub genes in both groups. In Figure 5A, the expression levels of these seven genes (*CD8A, CD2, GZMB, CCL5, CXCL9, IL7R, CD27*) were shown. In RA joints, *CD8A, CD2, GZMB, CCL5, CXCL9, IL7R,* and *CD27* levels were significantly higher than in OA joints. Further, IHC was used to determine the expression levels of seven different hub genes in large and small joints of RA and OA joints. As shown in Figures 5B, C, we found the protein expressions of CD8A, GZMB, CCL5, CD2, and CXCL9 were significantly higher in RA-LJ than that in OA, while the protein levels of CD8A, CD2, IL7R, CD27, and GZMB in RA-SJ were significantly higher than in OA joint (Figures 5D–F).

## Discussion

Rheumatoid arthritis and osteoarthritis are two of the most common of the more than 100 different types of arthritis. As a result of chronic inflammation and autoimmunity associated with RA disease, synovial fluid is produced by the membrane lining, causing synovitis, joint pain, and eventually chronic and progressive erosion (Imas et al., 2020). As a whole-joint disease, OA causes increased remodeling of cartilage, subchondral bone, bone marrow, and synovium, as well as the joint (Hügle and Geurts, 2017). For proper treatment, it is necessary to understand the different mechanisms of the two diseases. As part of this study, we compared gene expression profiles in synovial tissue between large and small RA joints and OA joints using bioinformatic analysis. By using the online tool GEO2R, differentially expressed genes were screened. In addition, we identified 5 DEGs that have the most interactions in the two PPI networks as hub genes of two comparison groups. In RA-LJ *versus* OA groups, *CD8A, GZMB, CCL5, CD2,* and *CXCL9* are the hub genes, while *CD8A, CD2, IL7R, CD27,* and *GZMB* are the hub genes in RA-SJ *versus* OA groups. Lastly, immunohistochemistry was used to validate the protein expression levels of the 7 hub genes in RA and OA joint tissues.

Based on our analysis, we found that GO enrichments were mainly related to T cell activation, B cell activation, chemokine activity, and collagen-containing extracellular matrix in large and small joints of RA. In RA joints, T cell receptor signaling pathways and cytokine-cytokine receptor interactions were the main signaling pathways. It is easy to understand from these results for that rheumatoid arthritis is an autoimmune and inflammatory disease, which also suggests different treatment strategies for RA and OA. Rituximab, a kind of anti-CD20 chimeric monoclonal antibody that can inhibit the proliferation of stimulated human B cells, has long been a choice for refractory rheumatoid arthritis (Tavakolpour et al., 2019). Moreover, bDMARDS are now widely used to treat RA. The majority of bDMARDS are anti-cytokine or anti-chemokine molecules, but some patients do not respond to existing bDMARDs. Therefore, new bDMARDs for RA need to be developed.

Hub genes *CCL5*, also known as *RANTES*, its protein is a chemoattractant for blood monocytes, memory T helper cells, and eosinophils. It causes the release of histamine from basophils and activates eosinophils and plays an active role in the chemotactic activity of T cells in RA by facilitating leukocyte infiltration (Luterek-Puszyńska et al., 2017). Solomon A. et al. have demonstrated that CCL5 induced a positive inflammatory response in RA by activating synovial fibroblasts, thereby facilitating matrix metalloproteinase-1 (MMP-1) and MMP-13-mediated destruction of the extracellular matrix (Agere et al., 2017). The pathological process of arthritis is also mediated by another gene, *CXCL9*, whose protein is detected in sera, synovial fluid, and synovial tissue (Lee et al., 2011; Yoshida et al., 2012; Pandya et al., 2017). By binding to CXCR3 on synovial tissue, inflammatory chemokines attract Th1 cells and macrophages, thereby contributing to arthritis accumulation. These findings suggest potential bDMARDS targets for treating RA in the future.

T cells participate in multiple pathways driving the disease process in rheumatoid arthritis. Naive CD4^+^ T cells from patients with RA transition into highly proliferative, tissue-invasive and proinflammatory effector cells (Weyand and Goronzy, 2017). Equipped with tissue-invasive features, RA CD4^+^ T cells rapidly induce synovitis in a human synovium mouse chimera model (Weyand and Goronzy, 2021). It was found that synovial T cells, together with synovial macrophages, are the cellular origin of TNF (Zhang et al., 2019). To date, however, anti-T cell therapy has not been one of the major success stories of RA. Because there is still a lack of a highly selected target for anti-T cell therapy, completely blocking T cell activity is not feasible. F Zhang et al. defined distinct subsets of CD8^+^ T cells characterized by GZMK+, GZMB+, and GNLY + phenotypes in RA joint synovial tissues by integrating single-cell transcriptomics and mass cytometry (Zhang et al., 2019), indicating an important role of hub gene *GZMB* in the classification of CD8^+^ T cells in RA. It has already been found for decades that there was a remarkable increase in GAMB concentrations in plasma and synovial fluid of patients with established rheumatoid arthritis compared with disease controls (Tak et al., 1999). Besides, IL-7/IL-7R physiologically promotes T cell proliferation and prolonged survival as well as pathologically influencing Th1/Th17 cell differentiation, potentiated glycolysis, and expansion of osteoclast maturation, which contributes to the neovascularization in RA synovial tissue (Meyer et al., 2022). In addition, CD27, also known as TNFRSF7, is constitutively expressed on most T cells, and the interaction with its ligand CD70, can provide signals to T cells to control their accumulation and reactivity (Croft and Siegel, 2017). It has been found for a long time that in the synovial fluid of patients with RA, soluble CD27 levels and CD27^+^ T cell numbers are elevated and correlate with the levels of rheumatoid factor, supporting a role for CD27 in human RA (Tak et al., 1996). Blocking CD27^−^CD70 interactions with anti-CD70 antibody reduces bone and cartilage erosion and inflammatory infiltrates in the joints of mice with collagen-induced arthritis, moreover, decreases collagen-specific antibody production (Oflazoglu et al., 2009). In summary, molecules like GZMB, IL-7R, and CD27 offer the possibility of highly targeted and sophisticated therapies for RA.

## Conclusion

In conclusion, we screened differentially expressed genes between large and small joints of RA between OA joints in this study. Several functional and pathway enrichments were also found between RA and OA joints, primarily related to T cell activation or chemokine activity. It was determined whether RA and OA joint samples expressed hub genes such as *CD8A, CD2, GZMB, CCL5, CXCL9, IL7R,* and *CD27*. As a result of our study, we may be able to identify new diagnostic markers and therapeutic targets for RA and OA.

## Data Availability

The datasets presented in this study can be found in online repositories. The names of the repository/repositories and accession number (s) can be found in the article/Supplementary Material.

## Supplementary material

SUPPLEMENTARY FIGURE S1 The correlation between hub genes and CRP, DAS28-CRP, and ACPA/RFpositivity.**(A)-(D)** The level of IL-7R was negatively correlated with the concentrationof CRP in RA-LJ. No hub gene has a significant correlation with the DAS28-CRP score. **(E)** The expression of CD2 in the ACPA/RF positive group was significantly higherthan that in ACPA/RF negative group.

SUPPLEMENTARY TABLE S1 DEGs of RA-LJ and OA.

SUPPLEMENTARY TABLE S2 DEGs of RA-SJ and OA.
