# Supplementary figures and images for: Identification of key candidate genes and pathways in rheumatoid arthritis and osteoarthritis by integrated bioinformatical analysis
Source: Front Genet. 2023 Feb 13;14:1083615. doi: 10.3389/fgene.2023.1083615 (PMC9968929; doi:10.3389/fgene.2023.1083615)

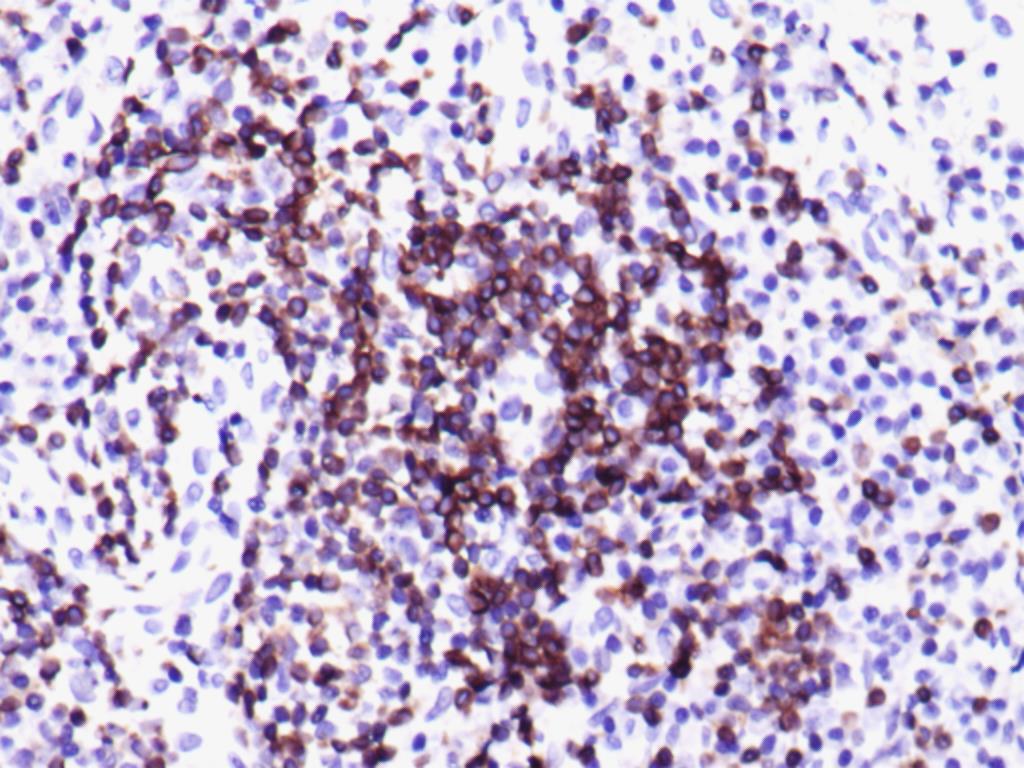

Supplement: Supplementary file 2 [file DataSheet4.ZIP › original microscopy images/CD2-RASJ.jpg]

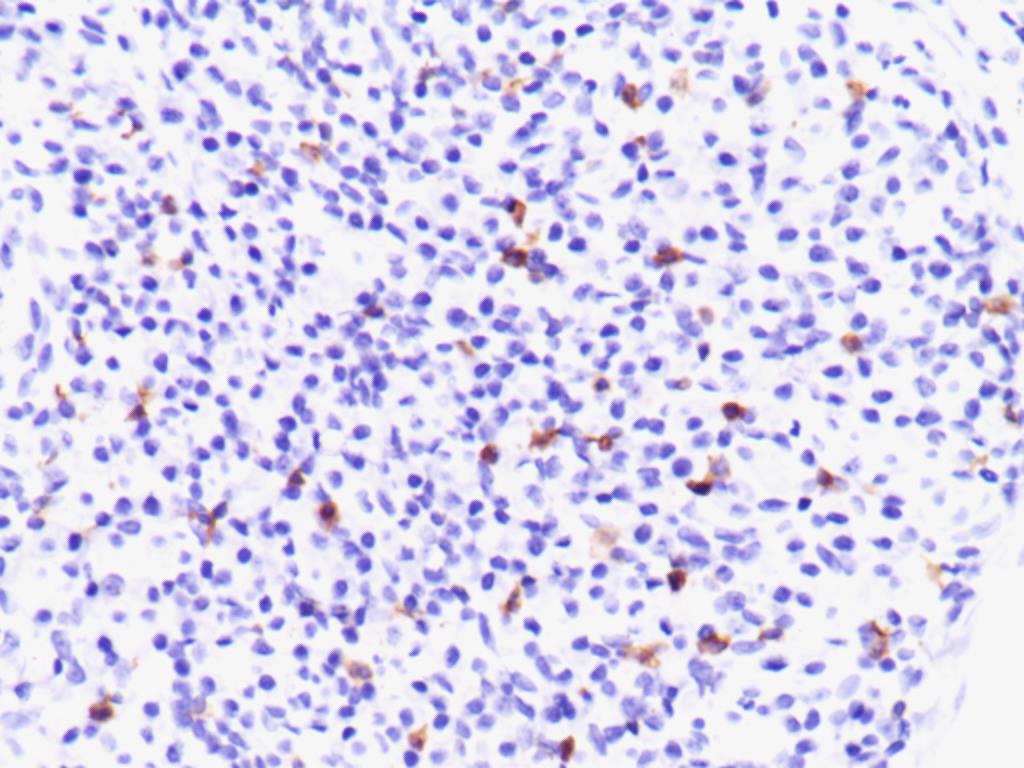

Supplement: Supplementary file 2 [file DataSheet4.ZIP › original microscopy images/OA-CCL5.jpg]

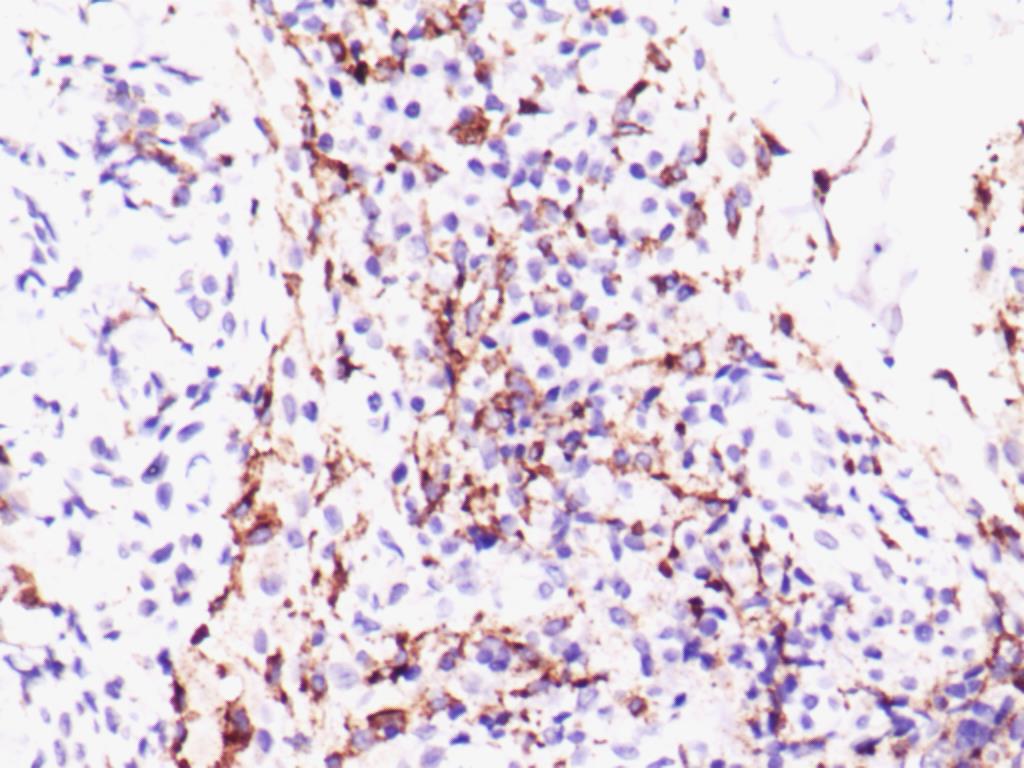

Supplement: Supplementary file 2 [file DataSheet4.ZIP › original microscopy images/GZMB-RASJ.jpg]

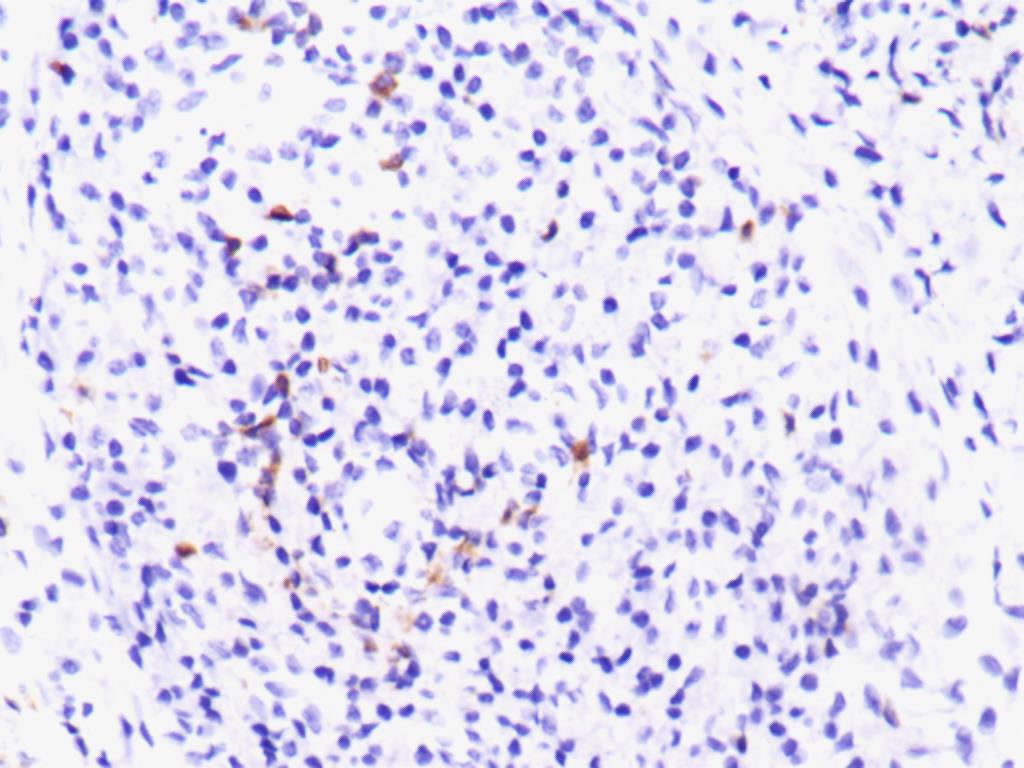

Supplement: Supplementary file 2 [file DataSheet4.ZIP › original microscopy images/OA-CXCL9.jpg]

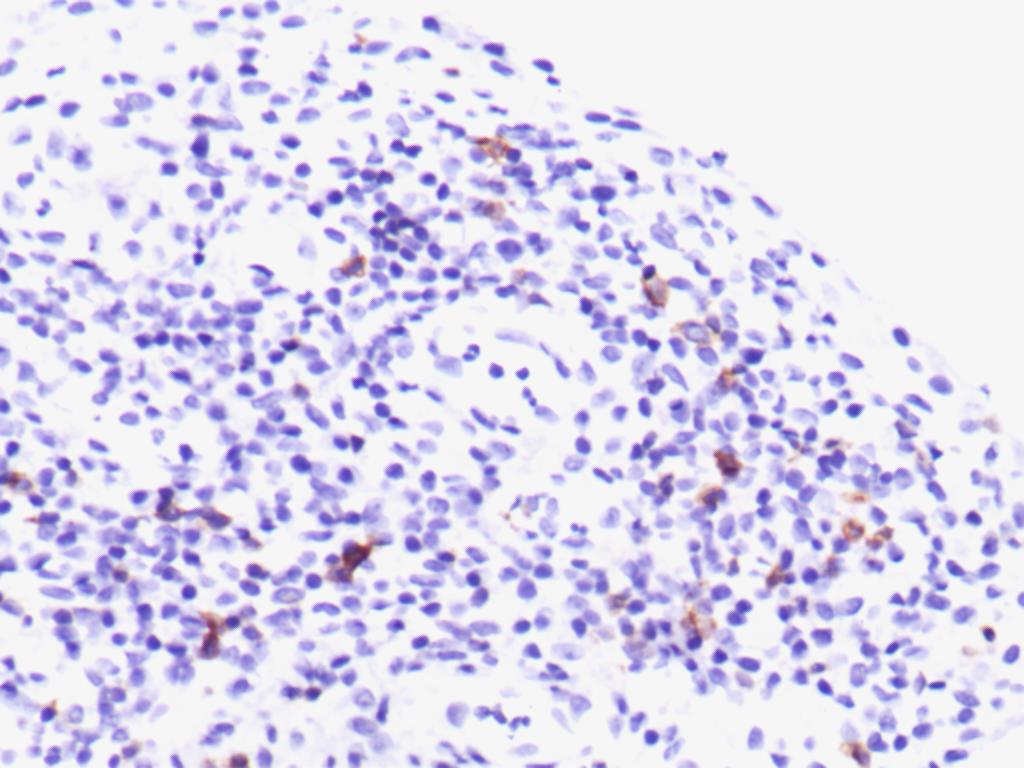

Supplement: Supplementary file 2 [file DataSheet4.ZIP › original microscopy images/CD8A-OA-2.jpg]

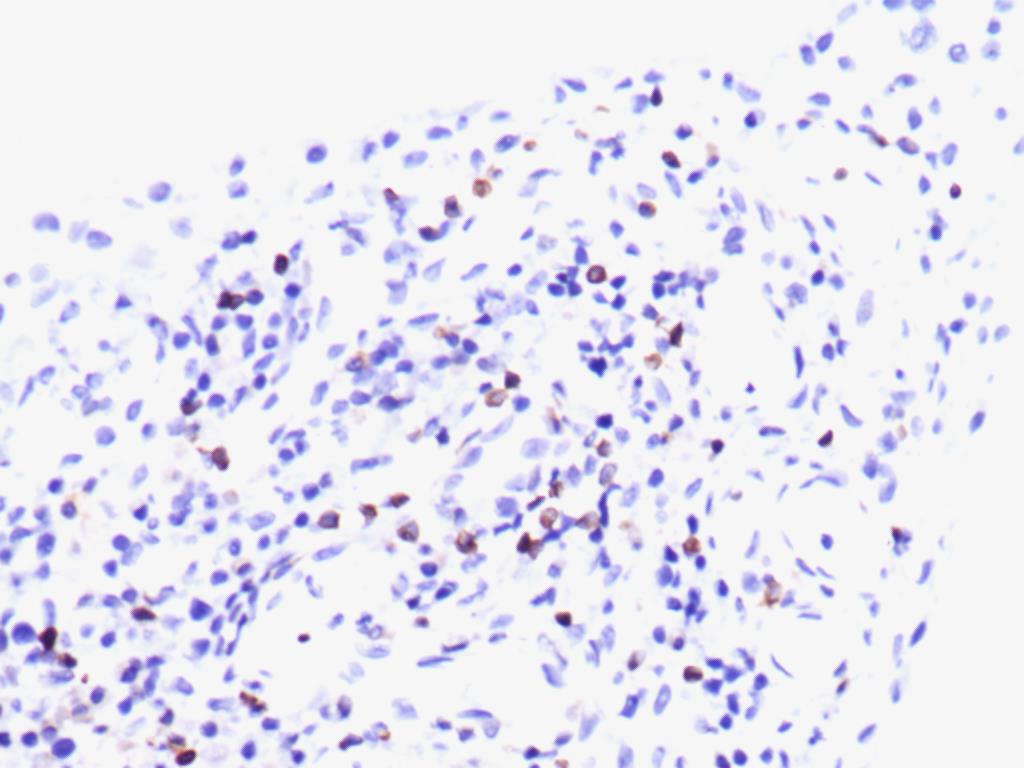

Supplement: Supplementary file 2 [file DataSheet4.ZIP › original microscopy images/CD8A-OA-1.jpg]

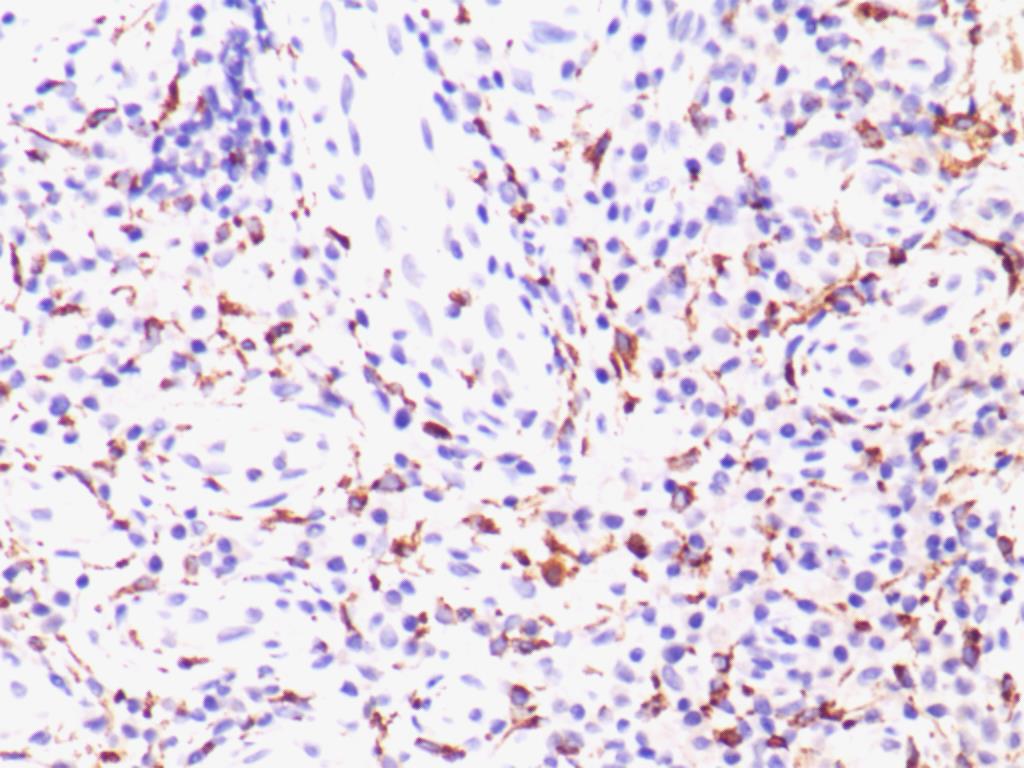

Supplement: Supplementary file 2 [file DataSheet4.ZIP › original microscopy images/RA-LJ-CXCL9.jpg]

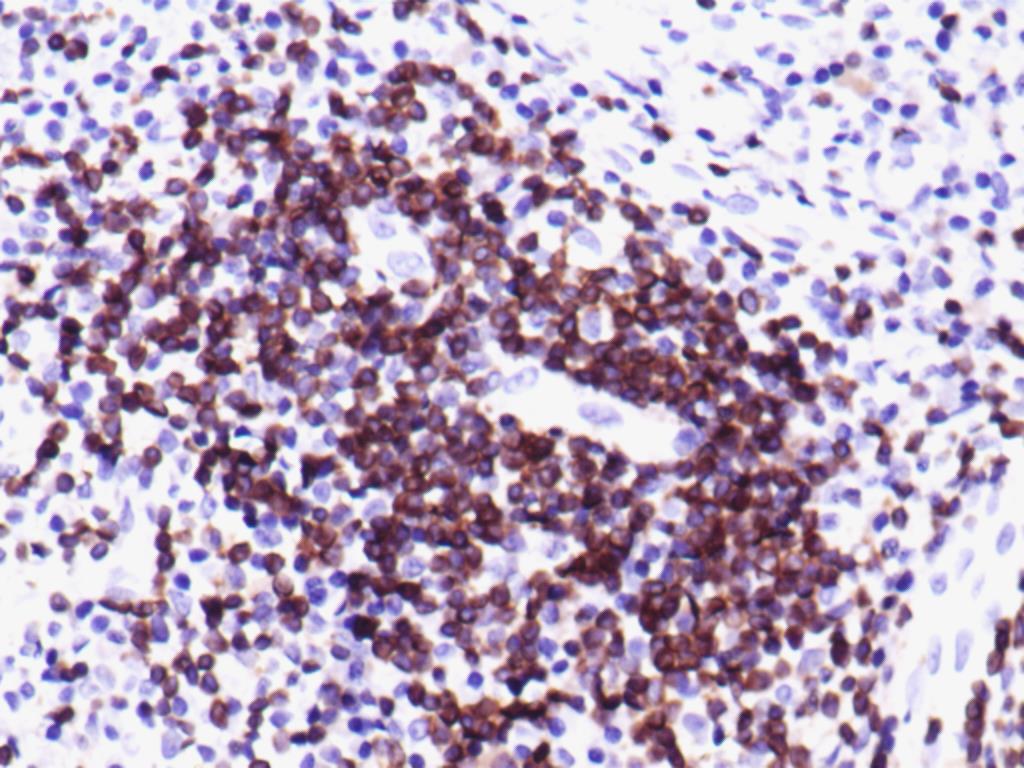

Supplement: Supplementary file 2 [file DataSheet4.ZIP › original microscopy images/CD2-RALJ.jpg]

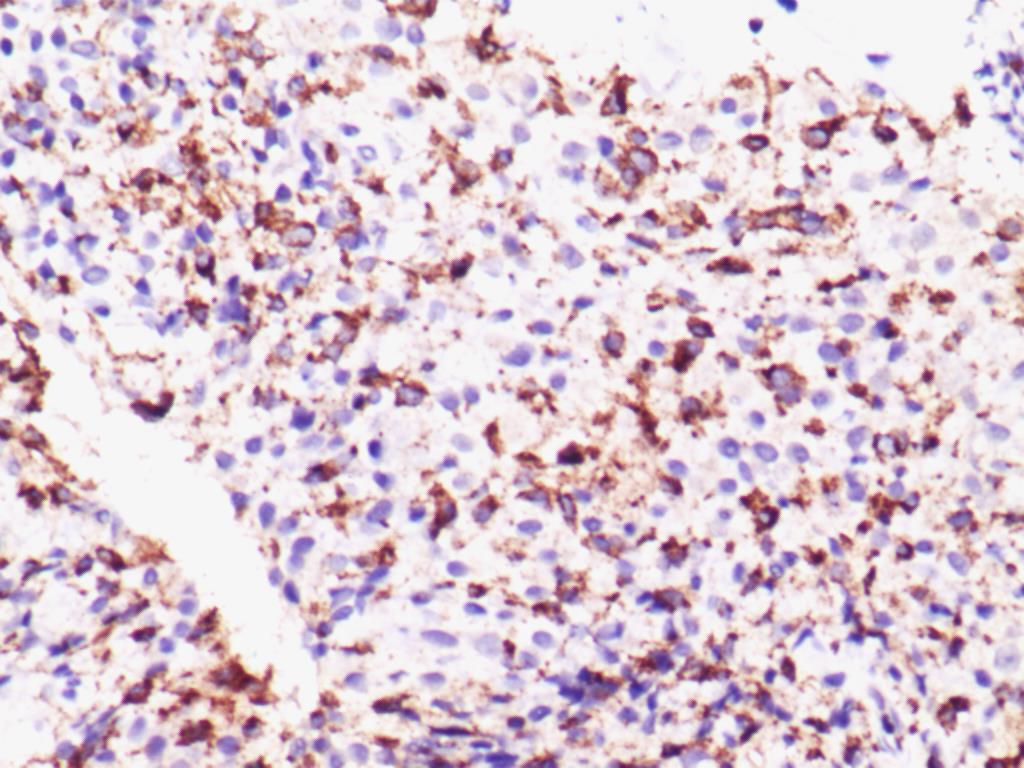

Supplement: Supplementary file 2 [file DataSheet4.ZIP › original microscopy images/GZMB-RALJ.jpg]

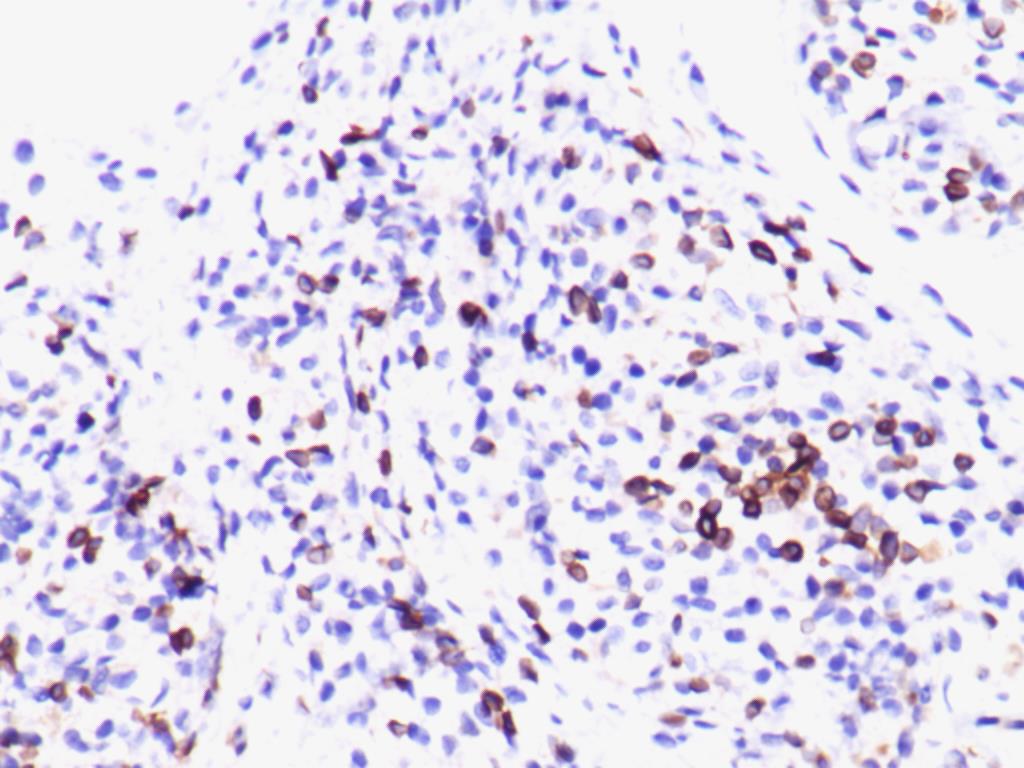

Supplement: Supplementary file 2 [file DataSheet4.ZIP › original microscopy images/RA-SJ-IL7R.jpg]

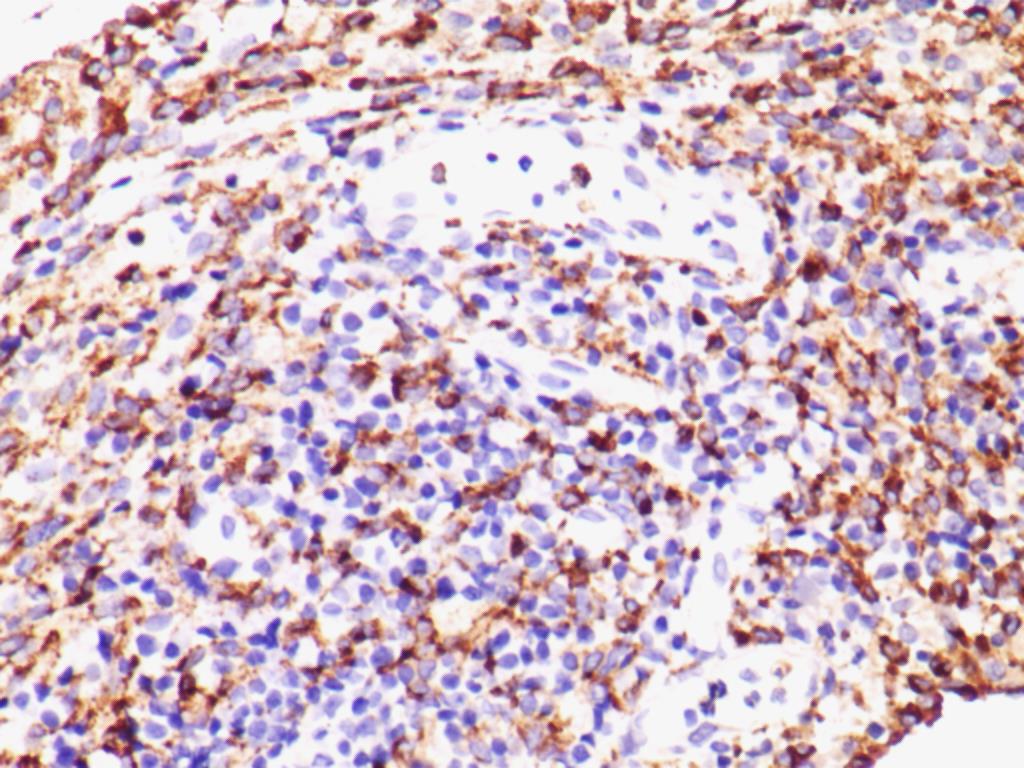

Supplement: Supplementary file 2 [file DataSheet4.ZIP › original microscopy images/RA-SJ-CD27.jpg]

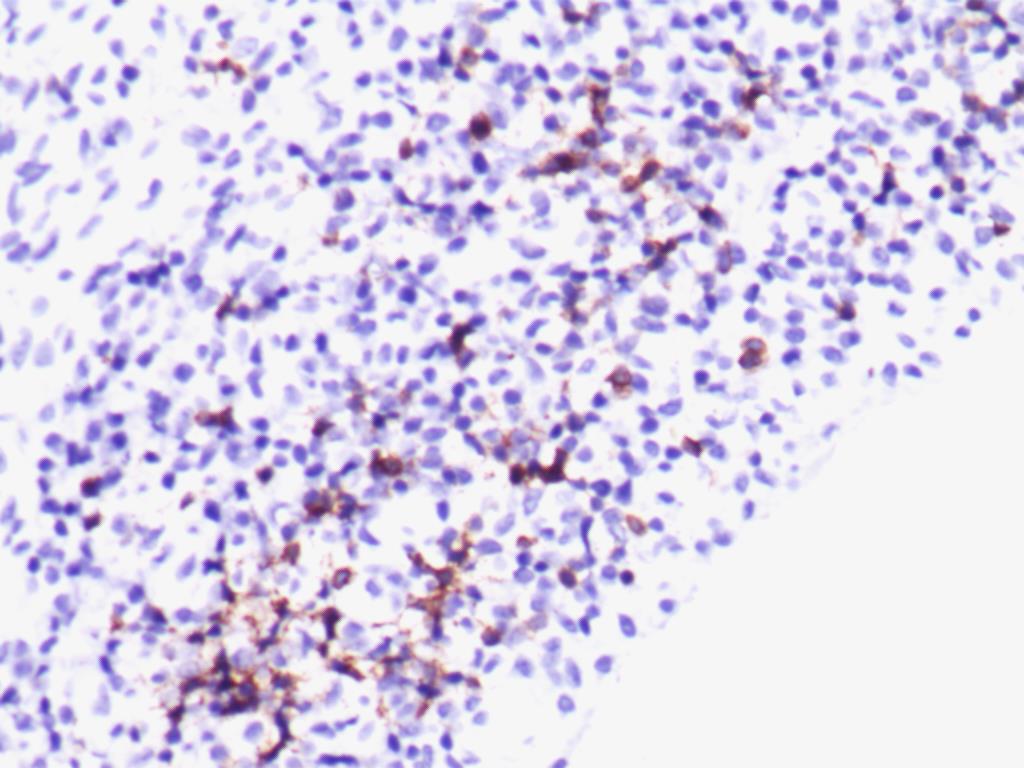

Supplement: Supplementary file 2 [file DataSheet4.ZIP › original microscopy images/CD8A-RASJ.jpg]

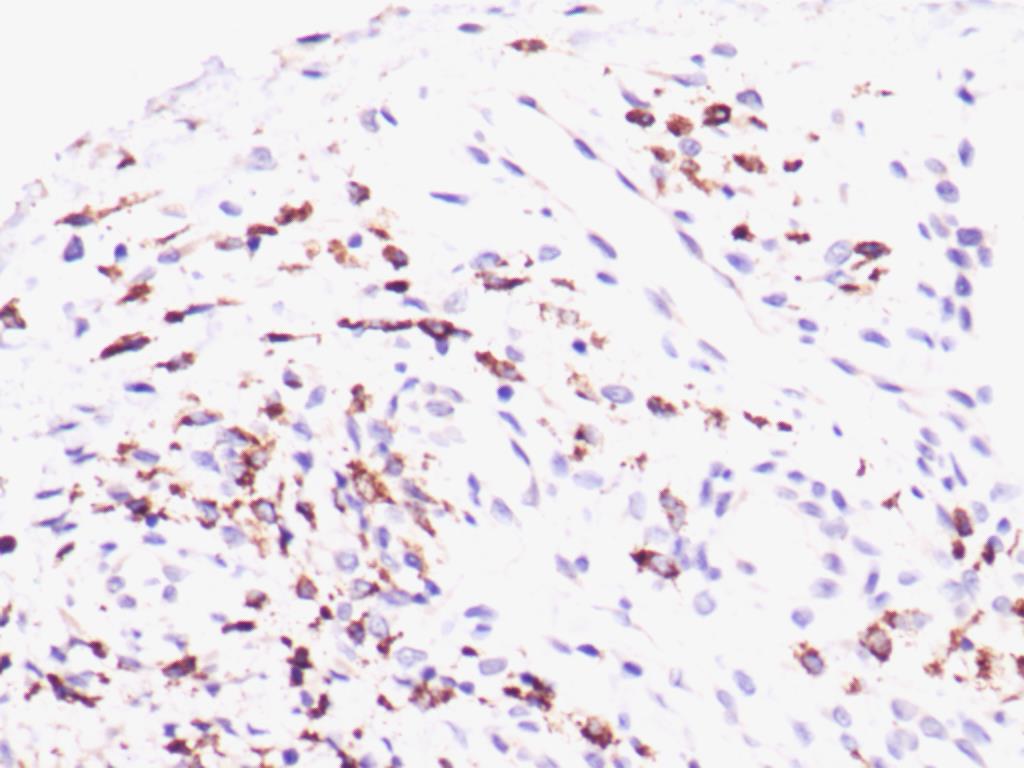

Supplement: Supplementary file 2 [file DataSheet4.ZIP › original microscopy images/GZMB-OA-2.jpg]

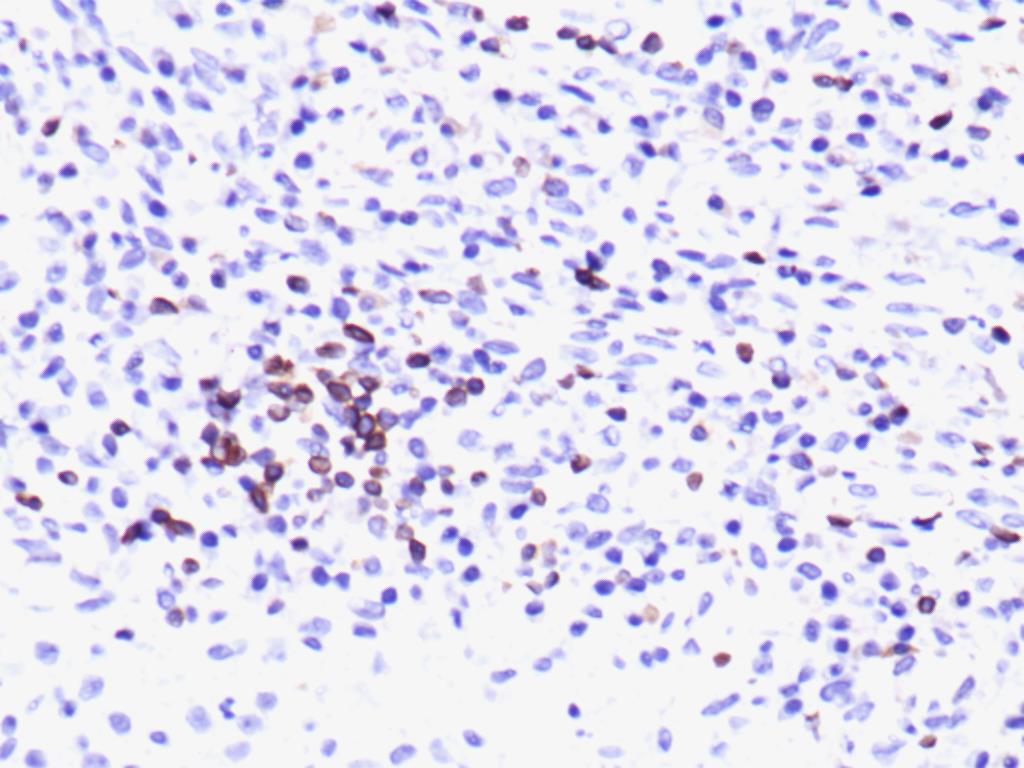

Supplement: Supplementary file 2 [file DataSheet4.ZIP › original microscopy images/CD2-OA-1.jpg]

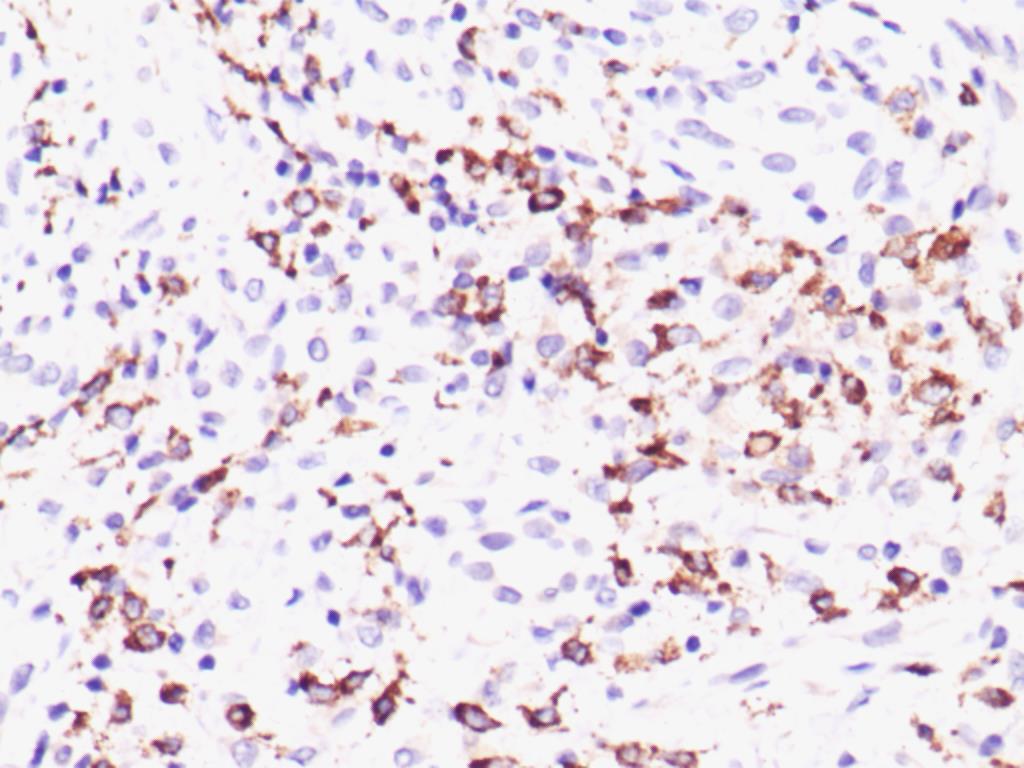

Supplement: Supplementary file 2 [file DataSheet4.ZIP › original microscopy images/GZMB-OA-1.jpg]

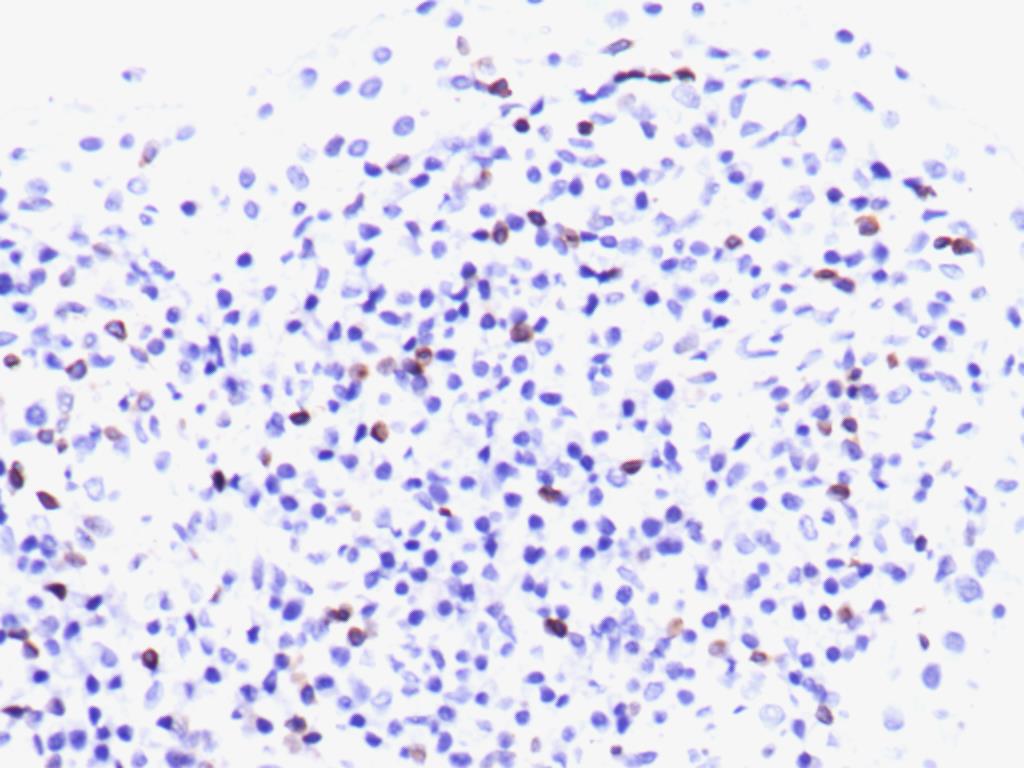

Supplement: Supplementary file 2 [file DataSheet4.ZIP › original microscopy images/CD2-OA-2.jpg]

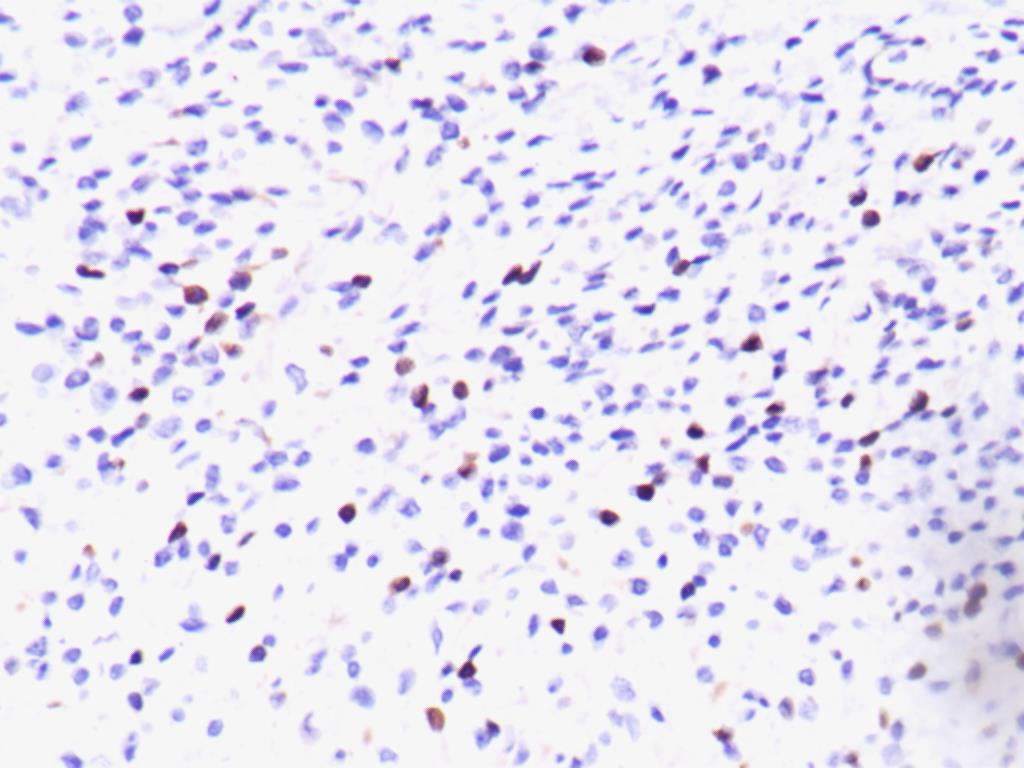

Supplement: Supplementary file 2 [file DataSheet4.ZIP › original microscopy images/OA-IL7R.jpg]

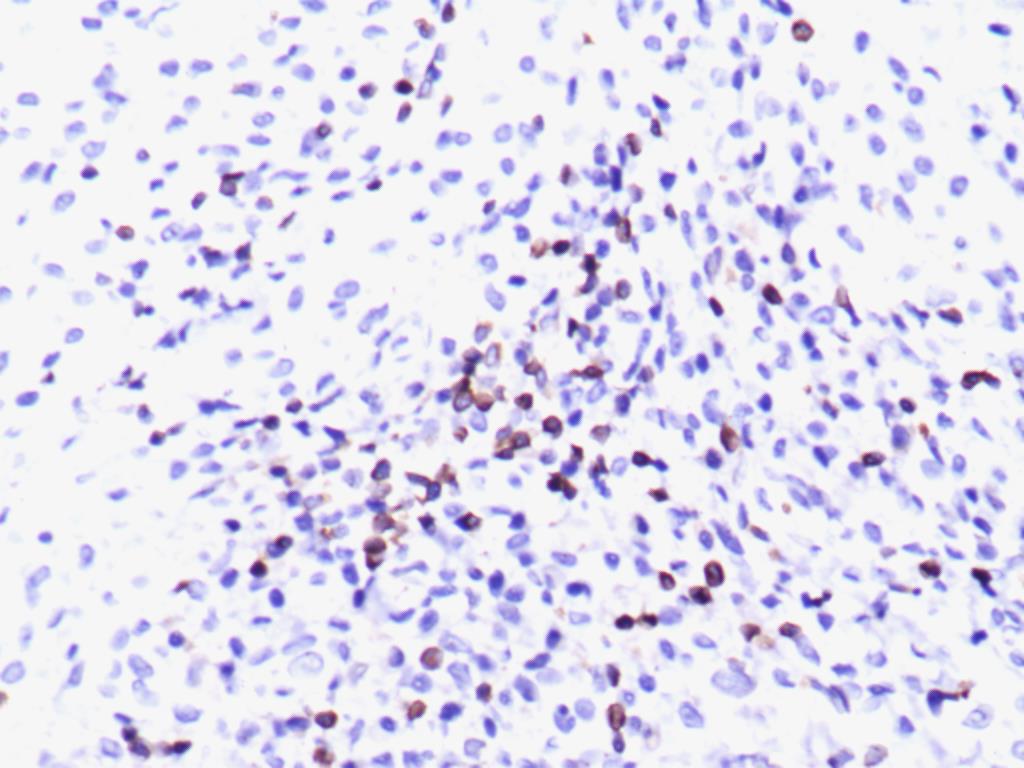

Supplement: Supplementary file 2 [file DataSheet4.ZIP › original microscopy images/CD8A-RALJ.jpg]

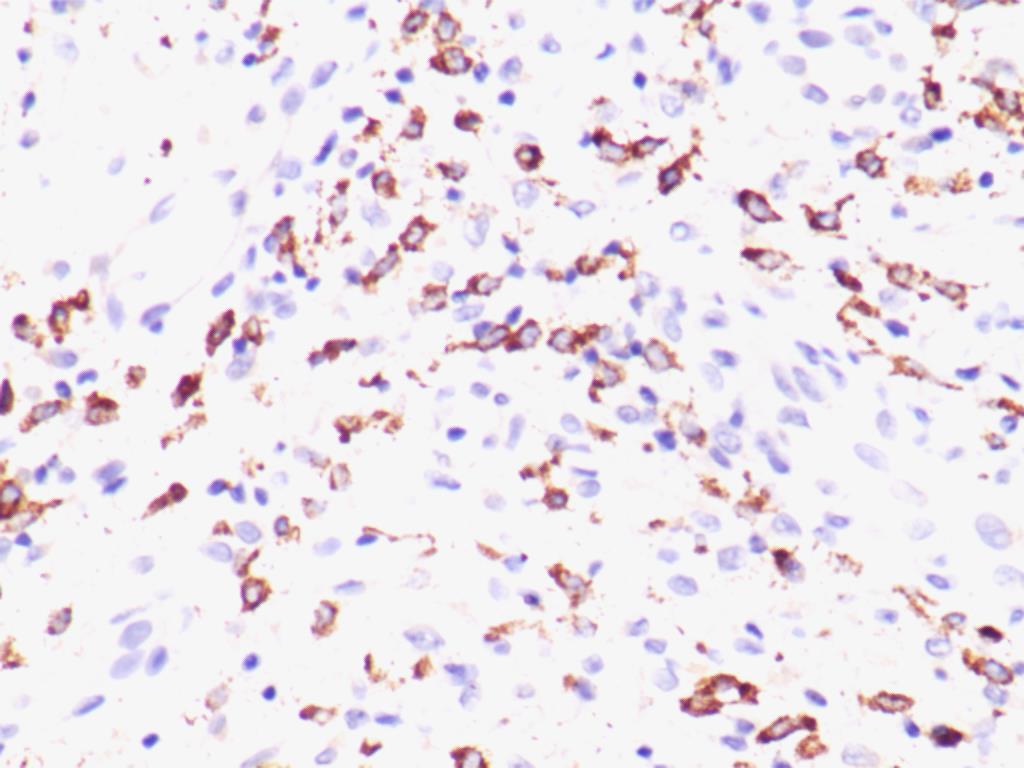

Supplement: Supplementary file 2 [file DataSheet4.ZIP › original microscopy images/OA-CD27.jpg]

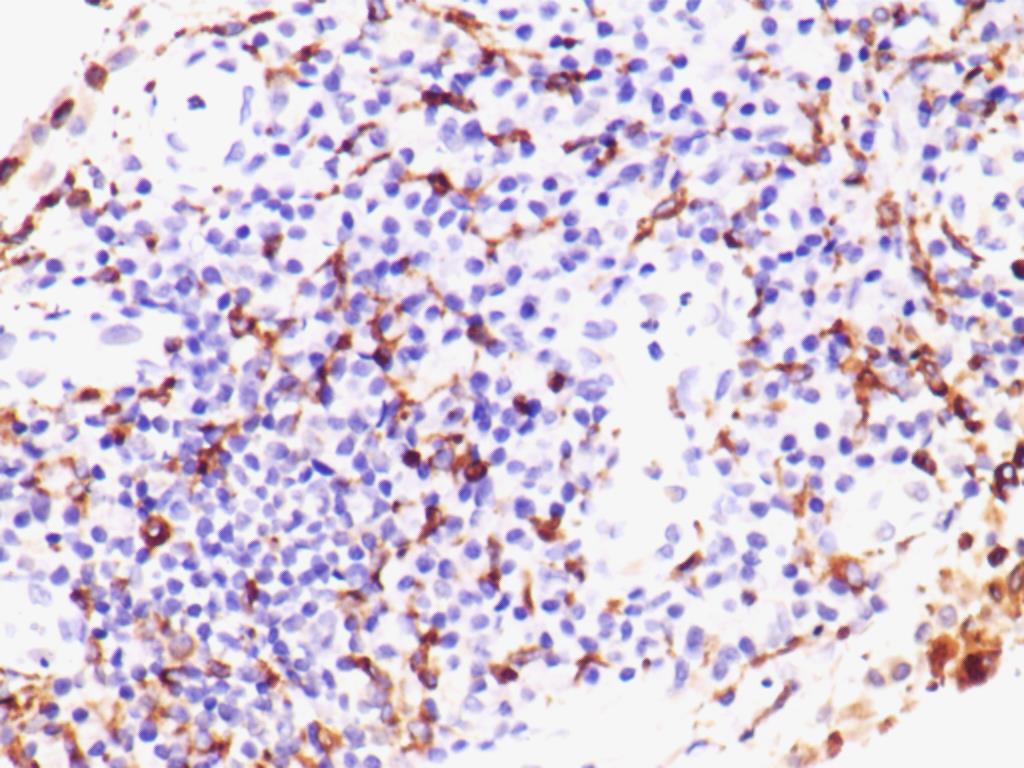

Supplement: Supplementary file 2 [file DataSheet4.ZIP › original microscopy images/RA-LJ-CCL5.jpg]

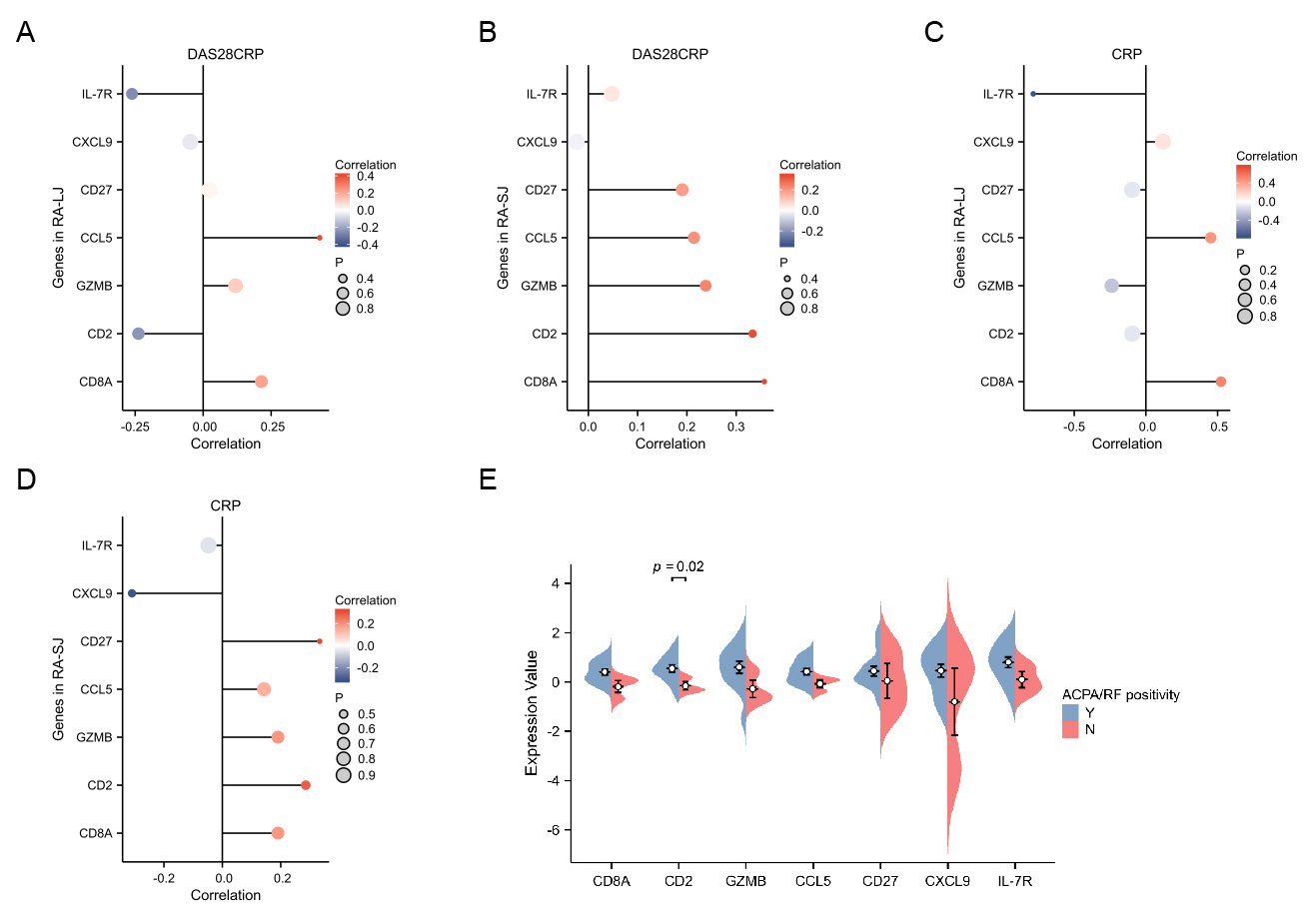

Supplement: Supplementary file 3 [file Image1.JPEG]
